# Lipophilicity and Pharmacokinetic Properties of New Anticancer Dipyridothiazine with 1,2,3-Triazole Substituents

**DOI:** 10.3390/molecules27041253

**Published:** 2022-02-13

**Authors:** Beata Morak-Młodawska, Małgorzata Jeleń

**Affiliations:** Department of Organic Chemistry, Faculty of Pharmaceutical Sciences in Sosnowiec, The Medical University of Silesia, Jagiellońska 4, 41-200 Sosnowiec, Poland; manowak@sum.edu.pl

**Keywords:** lipophilicity, RP-TLC, ADME properties, dipyridothiazine, 1,2,3-triazole, anticancer activity, Lipinski’s, Ghose’s, Veber’s rules

## Abstract

The lipophilicity parameters (log*P_calcd_*, *R_M_*_0_ and log*P_TLC_*) of 10 new active anticancer dipirydothiazines with a 1,2,3-triazole ring were determined theoretically using computational methods and experimentally by reversed-phase TLC. Experimental lipophilicity was assessed using mobile phases (a mixture of TRIS buffer and acetone) using a linear correlation between the *R_M_* retention parameter and the volume of acetone. The *R_M_*_0_ parameter was correlated with the specific hydrophobic surface b, revealing two congenerative subgroups: 1,2,3-triazole-1,6-diazaphenothiazines and 1,2,3-triazole-1,8-diazaphenothiazines hybrids. The *R_M_*_0_ parameter was converted into the log*P_TLC_* lipophilicity parameter using a calibration curve. The investigated compounds appeared to be moderately lipophilic. Lipophilicity has been compared with molecular descriptors and ADME properties. The new derivatives followed Lipinski’s, Ghose’s and Veber’s rules.

## 1. Introduction

The lipophilicity of compounds allows for the prediction of a compound’s fate in living organisms and indicates the types of transport and accumulation of the drug in the body. Lipophilicity is useful as an essential property of drugs at the time of their design so as to obtain the optimal properties required to achieve a molecular target [1,2]. The knowledge of this parameter is extremely important in metabolic transformations with the participation of bioactive molecules and their affinity for the protein target. Lipophilicity is believed to regulate the transport of a biologically active substance in its environment. Therefore, optimization of lipophilicity allows us to find the optimal drug structure in terms of quantification, structure-activity relationship studies (QSAR) [3,4,5].

**Figure 1:**
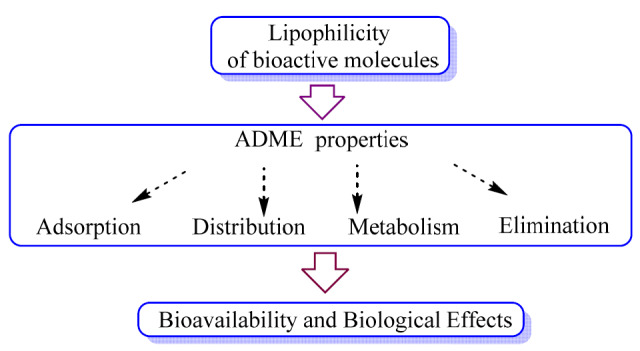
Influences of lipophilicity on ADME properties and final biological effects.

The definition of IUPAC shows lipophilicity as the affinity a molecule or moiety has for a lipophilic or non-polar environment [6]. Additionally, lipophilicity is one of the fundamental properties of compounds required to assess absorption, distribution, metabolism, and elimination (ADME parameters) in biological systems, in addition to their solubility, stability, and acid-base nature (Figure 1). Before the molecule reaches its pharmacological target, the lipophilicity of a compound indicates that the structure is similar to its lipophilic environment, allowing it to be transported across protein–lipid membranes into the biological system, forming complexes between the compound and the receptor binding site [7,8].

Lipophilicity also belongs to one of the factors determining the bioavailability of the drug in Lipinski’s, Ghose’s, and Veber’s rules [9,10,11,12]. 

Dipyridothiazines are modified phenothiazine structures into which two pyridine rings have been introduced instead of two benzene rings [13]. In recent years, significant and highly promising anticancer activities of these heterocyclic systems have been proven [14,15,16,17]. Additionally, selected derivatives of this group showed immunomodulatory and antioxidant potential [18,19]. The biological activity of selected dipyridothiazines has been shown to depend on lipophilicity and in some way correlates with ADME parameters [20,21,22].

Recently, the synthesis of dipyridothiazine derivatives with 1,2,3-triazole substituents (these being 1,2,3-triazole-dipyridothiazine hybrids) and their promising anticancer activities have been published [23]. These compounds showed in vitro anticancer activity against cancer cell lines: glioblastoma SNB-19, colorectal carcinoma Caco-2, lung cancer A549, and breast cancer MDA-MB231. In our research, dipyridothiazine hybrids were divided into two batches: the first containing 2,7- and 3,6-diazaphenothiazines in their structure, and the second containing 1,6- and 1,8-diazaphenothiazines in their structure. Thorough tests of lipophilicity and ADME parameters were performed for both groups. The results of the first part of the study show the influence of the above parameters on activity [24]. 

**Figure 2:**
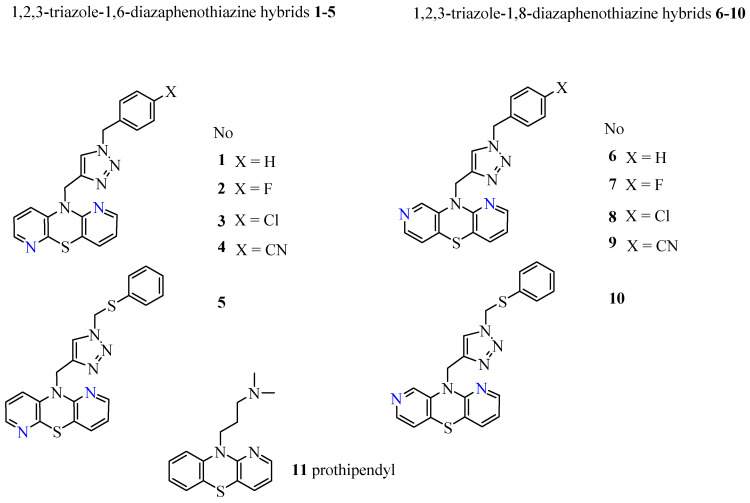
Structure of novel 1,6- and 1,8-diazaphenothiazine with 1,2,3-triazole substituents (**1**–**10**) and reference compound prothipendyl (**11**).

The results presented in this paper are a continuation of previous research [24] focused on 1,6- and 1,8-diazaphenothiazine derivatives. We investigated the lipophilicity of two series of 1,2,3-triazole-1,6-diazaphenothiazine (**1**–**5**) and 1,2,3-triazole-1,8-diazaphenothiazine (**6**–**10**) hybrids by RP TLC methodology, calculated programs, and studying the established relationships between their lipophilicity and ADME properties. The structures of the investigated compounds are presented in Figure 2. The lipophilicity was studied with the intention that it would provide a better insight into the differences in biological activity and also to deeper trace the influence of lipophilicity in reaching a molecular target.

## 2. Results

In the first stage of the study, eleven popular computer programs (VCCLAB and SwissADME [25,26,27,28]) based on different algorithms were used. The log*P_calcd_* values for the substituted dipirydothiazine-1,2,3-triazole hybrids **1**–**10** were different depending on the substituents in 1,2,3-triazole rings, places of nitrogen atoms in the dipyridothiazine system, and on the program used. The log*P_calcd_* values varied significantly from 2.08 to 5.09 (Table 1). The highest lipophilicity in the group of 1,6-diaphenothiazine derivatives was demonstrated according to the ALOGP module for compound **3** (log*P_calcd_* = 5.09) with a *p*-chlorophenyl substituent in its structure. On the other hand, the lowest lipophilicity in this group was calculated for derivative **4** with a *p*-cyanobenzyl (log*P_calcd_* = 2.08) according to the MLOGP program. Both of these programs predicted similar results in the 1,8-diazphenothiazine group, where the highest lipophilicity characterized compound **8** with a *p*-chlorobenzyl (log*P_calcd_* = 4.55), and the lowest derivative **9** with a *p*-cyanobenzyl (log*P_calcd_* = 2.08).

In further research, in order to obtain reliable values, the relative lipophilicities of derivatives **1**–**10** expressed by the chromatographic values of *R_M_*_0_ were measured by the experimental RP-TLC method. 

The experimental RP TLC method provided the retention parameter *R_M_* (calculated from the R_F_ values) using the following equation:

*R_M_* = log(1/R_F_−1)

The values of *R_M_* decreased linearly, with an increasing concentration of acetone in the mobile phase (*r* = 0.9885–0.9981). The extrapolation to 0% concentration of acetone gave the relative lipophilicity parameter (*R_M_*_0_) values, which showed the partitioning between the non-polar stationary and polar mobile phases, using the equation:

*R_M_* = *R_M_*_0_ + *b*C

where C is the concentration of acetone. The *R_M_*_0_ values were found to be within the range of 1.975–2.701 (Table 2).

The presented 1,2,3-triazole and dipyridothiazine hybrid derivatives **1**–**10** belong to another group of isomeric dipyridothiazines of structure 1,6- and 1,8-diazaphenothiazines. Therefore, they are isomers of the hybrids described above [24]. Structurally, they differ only in the location of nitrogen atoms in the azaphenothiazine core. These compounds do not show substantial differences in molecular descriptors, nevertheless the ADME parameters are substantially different (Table 3 and Table 4). All tested derivatives meet the requirements of Lipinski’s rule of five as well as the rules of Ghose and Veber [27] (Table 3).

In order to determine the pharmacokinetic properties of the tested group of compounds, the PreADMET server was used to calculate the following parameters: BBB, Caco-2, HIA, MDCK, PPB and SP (Table 4) [29]. Caco-2 and MDCK (Madin-Darby dog kidney) cell models have been calculated and are recommended as highly reliable in vitro models for predicting oral drug absorption. Another in silico human intestinal absorption (HIA) and skin permeability (SP) model predicts and identifies potential drugs for oral and transdermal administration. The parameter BBB (blood–brain barrier penetration) informs about the possibility of the compound acting in the central nervous system, and the PPB model (binding plasma proteins) indicates the binding efficiency [30,31]. These studies also used prothipendyl, a weak centrally acting neuroleptic, as the reference compound. The values of the *R_M_*_0_ parameter were correlated with molecular descriptors and ADME activities (Table 5)

Then a calibration curve was created using analogous measuring conditions. The set of reference substances **A**–**E** with literature values of log*P_lit_* were used in the range of 1.21–3.54 (Table 6). This curve made it possible to convert the values of the relative lipophilicity parameter *R_M_*_0_ of the tested hybrids into the value of the absolute lipophilicity parameter log*P_TLC_*.

The log*P_TLC_* values for all new anticancer hybrids (**1**–**10**) are collected in Table 7. 

## 3. Discussion

This work focuses on the assessment of the lipophilicity of new, anticancer active dipyridothiazines linked to the 1,2,3-triazole ring (**1**–**10**), which are recognized in chemical literature as hybrids of both heterocycles. Two series of dipyridothiazines (1,6- and 1,8-diazaphenothiazines) contain a 1,2,3-triazole ring in which various benzyl substituents and a phenylthiomethyl substituent have been introduced (Figure 2).

These compounds showed promising anticancer activity in vitro against the tumor cell lines SNB-19 glioblastoma, Caco-2 colorectal carcinoma, A549 lung carcinoma and MDA-MB231 breast cancer, and low cytotoxicity against NHDF normal human fibroblasts. This group included derivatives **3** and **8** with *p*-chlorobenzyl substituents that showed highly promising activities against Caco-2, MDA-MB231 and A549 (IC_50_ in the range of 0.25–0.51 μM) [23]. The most active derivative, **3,** was analyzed for the expression of genes influencing the neoplastic process (*H3*, *TP53*, *CDKN1A*, *BCL-2* and *BAX*). These studies have shown the activation of the mitochondrial apoptosis pathway and disruptions in the proper formation of DNA histones [23].

We started our research with in silico lipophilicity calculations using the available VCCLAB and SwissADME internet servers. The calculated lipophilicity within these modules varies greatly, which is most likely related to the different mathematical models used to calculate it. 

**Figure 3:**
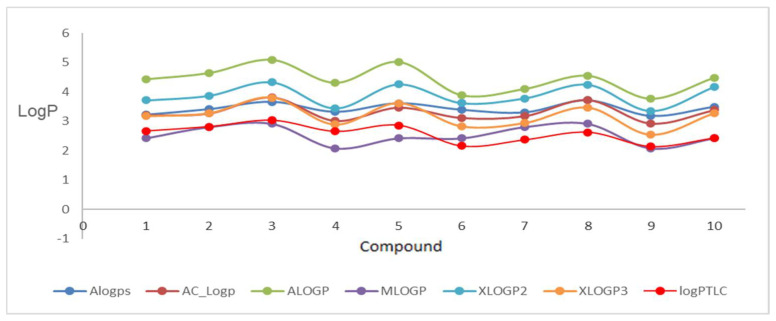
Graphical visualization of calculated log*P* values (using VCCLAB models) of the tested compounds with comparison of log*P_TLC._*

**Figure 4:**
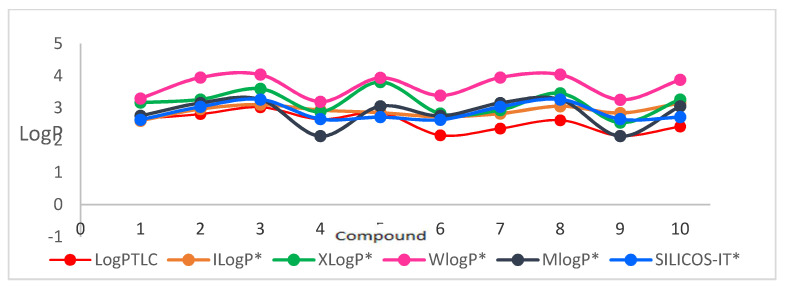
Graphical visualization of calculated log*P* values (using SwissADME models) of the tested compounds with comparison of log*P_TLC._* * results obtained using the SwissADME program.

The most lipophilic compound was derivative **3** (log*P_calcd_* = 5.09), but the isomeric compound **10** (log*P_calcd_* = 4.55) was slightly less lipophilic, both with a *p*-chlorobenzyl substituent at the triazole ring. The least lipophilic compounds were compound **4** and **9** (log*P_calcd_* = 2.08), which are isomers and contain a *p*-cyanobenzyl substituent in their structure. The results of these measurements are summarized in Table 1, and the graphical visualization of the calculated log*P* values of each compound is shown in Figure 3 and Figure 4. In the studies, large differences of over two units were observed for each compound. The most inflated results for the studied group of derivatives were indicated by the ALOGP program. Such large discrepancies in results were observed in our previous studies related to 2,7-diaza- and 3,6-diazaphenothiazines derivatives [22,23,34]. It is also an indication of the need to perform experimental measurements in order to correctly and accurately determine the lipophilicity parameter.

In the next stage of the research, we started to determine the relative lipophilicity parameter of *R_M_*_0_ according to the procedure described in chapters two and four. The highest relative value of lipophilicity *R_M_*_0_ was characteristic for compound **3** (with a *p*-chlorobenzyl substituent in the 1,2,3-triazole ring and in 1,6-diazaphenothiazine) (*R_M_*_0_ =2.872). Interestingly, isomer **8** (1,8-diazaphenothiazin) showed lower lipophilicity (*R_M_*_0_ =2.464). It should be noted that in the 1,8-diazaphenothiazines group this compound was the most lipophilic among all derivatives. Compound **9** (with a *p*-cyanobenzyl substituent) from the 1,8-diazaphenothiazine series was characterized by the least lipophilic character. 

It can be seen that all the isomeric 1,8-derivatives **6**–**10** exhibit substantially lower relative lipophilicity parameters (Table 2).

The interdependence between the relative lipophilicity parameter *R_M_*_0_ and the specific hydrophobic surface *b* for all compounds **1**–**10** is given by the equation:

*R_M_*_0_ = −82,626*b* − 0.5023 *r* = 0.9538

This relationship indicated the existence of structurally expected congeneric subgroups:

the 1,6-diazaphenothiazine derivatives **1**–**5** *R_M_*_0_ = −58.614*b* + 0.4567 *r* = 0.9741.
the 1,8-diazaphenothiazine derivatives **6**–**10** *R_M_*_0_ = −98.997*b* − 1.0412 *r* = 0.9781.

These relationships are closely related to the location of nitrogen atoms in the dipyridothiazine system. Similar situations were previously observed for hybrids of isomeric 2,7- and 3,6-diazaphenothiazines [24]. 

A calibration curve was performed to determine the absolute lipophilicity parameter log*P*. The standard substances were compounds with the known log*P* parameter: acetanilide, acetophenone, 4-bromoacetophenone, benzophenone, and antracene for which in the literature, log*P_lit_* values are in the range 1.21–5.53 (Table 6) [32,33].

The relative lipophilicity parameter *R_M_*_0_ for the reference substance was determined under the same conditions as for hybrids **1**–**10**.

The standard curve equation is as follows:

*logP_TLC_* = 0.9862*R_M0_* + 0.1957 (*r* = 0.9949, *s* = 0.2246, F = 359.97, *p* = 0.0002)

On the basis of the calibration curve, the absolute *logP_TLC_* parameter of all tested compounds **1**–**10** was determined. They fall within the scope of: 2.159–3.027 (Table 7).

Compound **3** was characterized by the highest lipophilicity, and the lowest for hybrid **6**. In the 1,6-diazaphenothiazines group, derivative **3** was the most lipophilic, whereas compound **4** was the least lipophilic. In the 1,8-diazaphenothiazines group, derivative **8** showed the highest lipophilicity and compound **9** the lowest. On this basis, it is noted that the *p*-chlorobenzyl substituent in both isomers increases the lipophilicity and the *p*-cyanobenzyl substituent lowers the lipophilicity.

**Figure 5:**
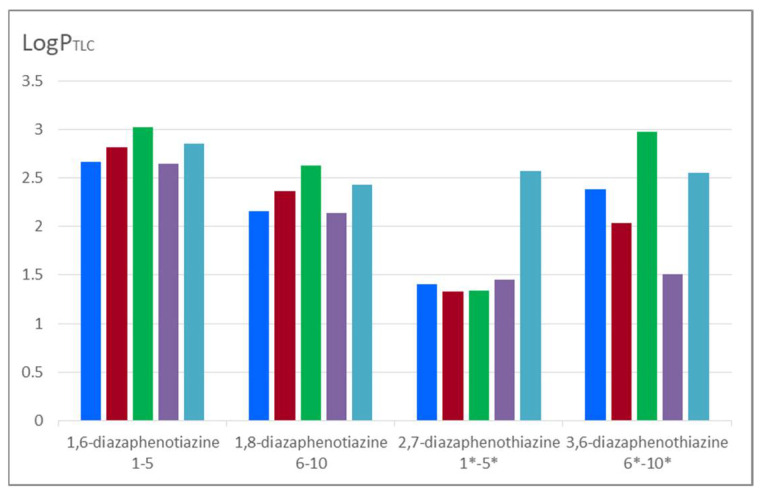
Graphical visualization of the experimental lipophilicity log*P_TLC_* values of the tested 1,6- and 1,8-diazaphenothiazine derivatives compared with the lipophilicity of previously described analogous 2,7- and 3,6-diazaphenothiazines [24]. The indicator * applies to derivatives 2,7-diazaphenothiazine and 3,6-diazaphenothiazine quoted from the publication [24].

Comparing the lipophilicity of the described 1,6- and 1,8-diazaphenothiazine derivatives **1**–**10** with the previously described group of 2,7- and 3,6-diazphenothiazine hybrids is illustrated in Figure 5. It can be noticed that the 2,7-diazaphenothiazine derivatives were the least lipophilic group of all isomers. Their lipophilicity was in the range of 1.408–2.569 [24]. It can be observed that the isomeric 1,6-diazaphenothiazines were characterized by the highest lipophilicity. It should be noted that in the group of tested compounds, the highest anticancer activity was demonstrated by the 1,6-diazaphenothiazine hybrid with a triazo ring and *p*-chlorobenzyl substituent **3** [23]. When these facts are compared with those of other isomeric hybrids, it can be assumed that this type of activity was not determined by lipophilicity.

Analysis of ADME parameters of compounds **1**–**10** compared with the reference compound **11** showed interesting information (Table 4). The tested compounds have BBB indices in the range of 0.352–2.156 which are substantially lower than those of reference compound **11** (3.103), which may indicate poor migration across the blood–brain barrier and low neurotoxicity. The permeability of Caco-2 cells was different among the tested derivatives. Compounds **1**, **2**, **4**, **6** and **7** have a comparable affinity to reference compound **11**. However, derivatives **3**, **5**, **8**, **9** and **10** were characterized by substantially higher indexes, which may indicate their stronger cellular affinity. All tested compounds exhibited a high HIA index, which was in the range of 97–99. The permeability of MDCK cells was variable and ranged from 1.78–48.87. Derivatives **1**–**5** exhibited lower parameters than derivatives **6**–**10**. The PPB parameter for the tested group of compounds is substantially higher than for the reference compound, which may indicate an increased ability to bind to plasma proteins. All the tested derivatives showed a poor SP index, which was comparable to the reference compound. The calculated ADME parameters showed the similarity of the tested derivatives to the drug substance.

In our research, we made attempts to correlate the relative lipophilicity parameter R_M0_ with molecular descriptors and ADME parameters (Table 5). These correlations showed moderate r values in the range of 0.3265–0.6892. These results may suggest that lipophilicity is one of the many factors directly influencing biological activity. Additionally, they may indicate that lipophilicity depends on the conformation of molecules, their ionic interactions or van der Walls interactions.

Moreover, all tested derivatives meet the requirements of Lipinski’s rule of five as well as the rules of Ghose and Veber, which point out that derivatives can become a drug with the ability for orally active use. The presented results are promising and encourage further continuation. 

## 4. Materials and Methods

### 4.1. Materials

The following reagents were used in the experimental studies to prepare the mobile phase: acetone (POCh, Gliwice, Poland), TRIS (tris (hydroxymethyl) aminomethane, Fluka). In order to prepare the calibration curve, five chemical compounds with the described lipophilicity parameter (log*P_lit_*) were used: acetanilide (A, 1.21 [32]), acetophenone (B, 1.58 [32]), 4-bromoacetophenone (C, 2.43 [33]), benzophenone (D, 3.18 [32]), antracene (E, 5.53 [32]). Dipyridothiaznine with 1,2,3-triazole substituents 1–10 were obtained in the reactions described earlier [23]. Prothipendyl (10-dimethylaminopropyl-1-azaphenothiazine) 11 (AWD Pharma, Radebeul, Germany) was used as the reference compound [24].

### 4.2. Chromatographic Procedure 

The experimental lipophilicity was determined using the RP-TLC method according to the reference [24]. Silica gel RP 18F_254S_ (Merck, Darmstadt, Germany) was used as a stationary phase and acetone and aqueous TRIS (tris(hydroxymethyl)aminomethane) buffer pH 7.4 was used as a mobile phase with a range from 40 to 70% (*v*/*v*), increased in 5% increments.

The compounds **1**–**11** and the standards **A**–**E** were dissolved in ethanol (2.0 mg/mL) and 2 μL of these solutions were spotted. Spots were observed under UV light at λ = 254 nm. Each measurement was performed in triplicate and then R_F_ values were calculated.

### 4.3. Computational Programs

The calculated lipophilicity was determined using various internet servers: VCCLAB [25] and SwissADME [27] including: Alogps, AC_Logp, ALOGP, MLOGP, XLOGP2, XLOGP3, ILopP, XlogP, WlogP, MlogP, SILICOS-IT. The molecular descriptor and parameters of Lipinski’s, Ghose’s and Veber’s rules were calculated using SwissADME server [27]. ADME parameters such as: human intestinal absorption (HIA), plasma protein binding (PB), blood–brain barrier (BBB), cell permeability (MDCK), skin permeability (SP), and Caco-2 penetration were calculated by PreADMET software [29].

## 5. Conclusions

The presented results show the lipophilicity of the isomeric dipyridothiazines (1,6- and 1,8-diazaphenothiazines) containing a 1,2,3-triazole ring in their structure. These compounds showed high anticancer potential in previous studies. The lipophilicity was determined theoretically by computational methods and experimentally with the use of reversed-phase thin-layer chromatography (RP TLC).

The test compounds were essentially more lipophilic than the previously described 2, 7- and 3,6-diazaphenothiazine derivatives with analogous substituents. Additionally, ADME parameters were determined, which were correlated in some way with lipophilicity. The new derivatives followed Lipinski’s, Ghose’s, and Veber’s rules, which is an indication that they may become orally administered drugs in the future. Subsequent studies of this group of compounds have been planned to fully define their pharmacological potential.

## Figures and Tables

**Table 1:** The calculated lipophilic parameters (log*P*_calcd_) for hybdrids of 1,2,3-triazole and dipyridothiazine **1**–**10** using internet data bases: VCCLAB and SwissADME * [25,27].

No	Alogps	AC_Logp	ALOGP	MLOGP	XLOGP2	XLOGP3	ILogP *	XLogP *	WlogP *	MlogP *	SILICOS-IT *
**1**	3.22	3.21	4.43	2.42	3.71	3.17	2.61	3.17	3.30	2.76	2.64
**2**	3.41	3.27	4.64	2.80	3.87	3.27	2.97	3.27	3.95	3.16	3.04
**3**	3.66	3.82	5.09	2.91	4.33	3.80	3.11	3.60	4.04	3.27	3.27
**4**	3.32	3.02	4.31	2.08	3.44	2.89	2.94	2.89	3.20	2.13	2.66
**5**	3.61	3.47	5.02	2.42	4.26	3.61	2.86	3.81	3.94	3.06	2.72
**6**	3.39	3.12	3.89	2.42	3.62	2.83	2.73	2.83	3.39	2.76	2.64
**7**	3.30	3.18	4.10	2.80	3.78	2.94	2.83	2.94	3.95	3.16	3.04
**8**	3.71	3.73	4.55	2.91	4.25	3.46	3.06	3.46	4.04	3.27	3.27
**9**	3.18	2.93	3.77	2.08	3.35	2.55	2.85	2.55	3.26	2.13	2.66
**10**	3.49	3.38	4.48	2.42	4.17	3.27	3.16	3.27	3.88	3.06	2.72

* results obtained using the SwissADME program.

**Table 2:** The *R_M_*_0_ values and *b* (slope) and *r* (correlation coefficient) of the equation *R_M_ = R_M0_* + *b*C for compounds **1**–**10**.

No	*−b*	*R_M0_*	*r*
**1**	0.0346	2.507	0.9946
**2**	0.0384	2.655	0.9951
**3**	0.0404	2.872	0.9932
**4**	0.0380	2.491	0.9981
**5**	0.0387	2.701	0.9946
**6**	0.0301	1.991	0.9908
**7**	0.0331	2.205	0.9925
**8**	0.0353	2.464	0.9885
**9**	0.0312	1.975	0.9895
**10**	0.0330	2.266	0.9899

**Table 3:** The molecular descriptor and parameters of Lipinski’s, Ghose’s and Veber’s rules for hybdrids of 1,2,3-triazole and dipyridothiazine **1**–**10** and prothipendyl **11**.

No	Molecular Mass (M)	H-Bond Acceptors	H-Bond Donors	Rotatable Bonds	TPSA	Lipinski’s Rules	Ghose’s Rules	Veber’s Rules
**1**	372	4	0	4	85.03	+	+	+
**2**	390	4	0	4	85.03	+	+	+
**3**	406	4	0	4	85.03	+	+	+
**4**	397	5	0	4	108.8	+	+	+
**5**	404	4	0	5	110.3	+	+	+
**6**	372	4	0	4	85.03	+	+	+
**7**	390	4	0	4	85.03	+	+	+
**8**	406	4	0	4	85.03	+	+	+
**9**	397	5	0	4	108.8	+	+	+
**10**	404	4	0	5	110.3	+	+	+
**11**	286	2	0	4	44.6	+	+	+

**Table 4:** The ADME activities predicted for 1,2,3-triazole-dipyridothiazine hybdrids **1**–**10** and prothipendyl **11**.

No	1	2	3	4	5	6	7	8	9	10	11
BBB	1.2664	1.6738	2.156	0.462	0.507	0.855	1.147	1.565	0.507	0.352	3.103
Caco-2	26.953	29.306	51.251	22.971	57.104	24.482	26.096	50.568	57.104	56.754	22.684
HIA	98.110	98.098	97.663	99.752	99.025	98.110	98.098	97.663	99.025	99.025	97.476
MDCK	31.186	4.540	16.317	9.067	1.787	48.877	7.323	19.818	1.787	1.930	18.983
PPB	95.034	94.700	97.370	91.793	91.234	92.088	91.670	94.175	91.234	90.008	75.453
SP	−3.328	−3.644	−3.378	−3.255	−3.189	−3.496	−3.802	−3.547	−3.189	−3.360	−3.100

**Table 5:** The correlation of the *R_M_*_0_ values with the molecular descriptors and predicted ADME activities for compounds **1**–**10**.

No	Molecular Descriptor or ADME Activities	Equation	*r*
**1–5**	M	*R_M_*_0_ = 8.424*M*^2^ + 104.7 *M* + 175.95	0.6791
**6–10**		*R_M_*_0_ = 9.5035*M*^2^ + 4.4337 *M* + 338.64	0.6892
**1–5**	TPSA	*R_M_*_0_ = −134.84*TPSA*^2^ + 697.89 *TPSA −* 805.1	0.3265
**6–10**		*R_M_*_0_ = −85.612*TPSA*^2^ + 36.19 *TPSA −* 282.83	0.4452
**1–10**	BBB	*BBB* = 0.6337 *R_M_*_0_^3^ − 10236 *R_M_*_0_^2^ + 0.1009*R_M_*_0_ + 2.3975	0.4732
**1–10**	Caco-2	*Caco*-2 = 0.6337 *R_M_*_0_^3^ − 1.0236 *R_M_*_0_^2^ + 0.1009*R_M_*_0_ − 2.3975	0.4732
**1–10**	HIA	*HIA* = −0.5781 *R_M_*_0_^3^ + 171.17 *R_M_*_0_^2^ − 1689*R_M_*_0_ + 55583	0.5626
**1–10**	MDCK	*MDCK* = 0.00006 *R_M_*_0_^3^ − 0.0011 *R_M_*_0_^2^ − 0.0378*R_M_*_0_ + 2.2632	0.6172
**1–10**	PPB	*PPB* = −0.0022 *R_M_*_0_^3^ + 0.6362 *R_M_*_0_^2^ − 60.72*R_M_*_0_ + 1930.3	0.6782
**1–10**	SP	*SP* = 0.818 *R_M_*_0_^3^ − 10.043 *R_M_*_0_^2^ − 39.971*R_M_*_0_ − 49.482	0.3793

**Table 6:** *R*_M0_ and log*P_lit_* values and *b* (slope) and *r* (correlation coefficient) of the equation *R*_M_ = *R*_M0_ + *b*C for standards **A**–**E**.

Parameters	A	B	C	D	E
log*P_TLC_*	1.21 [32]	1.58 [32]	2.43 [33]	3.18 [32]	5.53 [32]
R_M0_	1.001	1.501	2.231	2.886	3.488
−*b*	0.018	0.019	0.033	0.034	0.044
r	0.9979	0.9974	0.9960	0.9944	0.9964

**Table 7:** The log*P_TLC_* values of investigated compounds **1**–**10**.

	No of Compounds									
	1	2	3	4	5	6	7	8	9	10
log*P****_TLC_***	2.668	2.814	3.027	2.652	2.859	2.159	2.369	2.625	2.142	2.429

## Data Availability

Not applicable.

## Sample Availability

Samples of the compounds **1–10** are available from the authors.
